# Myelodysplastic Syndrome With Isolated del(5q) Coexisting With Cold Agglutinin Disease Presenting as Unexplained Hyperbilirubinemia

**DOI:** 10.7759/cureus.117773

**Published:** 2026-10-11

**Authors:** Madison N Sheppard, Lindsey Mayer, Shinoj Pattali Jayavalsan

**Affiliations:** 1 Medicine, Lake Erie College of Osteopathic Medicine, Bradenton, USA; 2 Hematology and Oncology, Ashtabula County Medical Center, Ashtabula, USA

**Keywords:** autoimmune hemolytic anemia (aiha), cold agglutinin disease, complement, del(5q), direct antiglobulin test, myelodysplastic syndrome (mds), sutimlimab

## Abstract

Cold agglutinin disease (CAD) is a rare autoimmune hemolytic anemia caused by cold-reactive IgM autoantibodies that activate the classical complement pathway. It is characteristically associated with clonal B-cell lymphoproliferative disorders, and its association with myelodysplastic syndrome (MDS) is rare. We report a 75-year-old woman evaluated over nine months for isolated, predominantly indirect hyperbilirubinemia attributed to hepatobiliary disease before hemolysis was recognized. An anti-IgG-only direct antiglobulin test (DAT) was negative, and she was referred as having Coombs-negative hemolytic anemia; a complement-inclusive DAT subsequently demonstrated polyspecific antiglobulin and anti-C3b/C3d positivity at 3+ with negative anti-IgG, with cold agglutinin titers above 1:1024 and low C4, confirming complement-mediated hemolysis. Recurrent specimen interference, including prewarming requirements and spuriously elevated mean corpuscular hemoglobin concentration, had been present for months. Bone marrow evaluation demonstrated MDS with isolated del(5q) alongside a small IgM-kappa monoclonal protein; to our knowledge, this coexistence has not previously been reported, although the source of the cold agglutinin was not established. After deterioration to a hemoglobin nadir of 7.3 g/dL, she received two units of packed red blood cells and the complement C1s inhibitor sutimlimab. Hemoglobin entered the reference range by day 33 and remained stable through day 103 without further transfusion, with near-normalization of lactate dehydrogenase and substantial improvement in bilirubin.

## Introduction

Cold agglutinin disease (CAD) is a rare form of autoimmune hemolytic anemia caused by cold-reactive immunoglobulin M (IgM) autoantibodies that bind erythrocytes at reduced temperatures and activate the classical complement pathway. Complement deposition on the erythrocyte surface drives hemolysis primarily within the reticuloendothelial system, producing the characteristic laboratory triad of anemia, elevated lactate dehydrogenase (LDH), and markedly decreased haptoglobin, accompanied by reticulocytosis [1,2]. Direct antiglobulin testing (DAT) characteristically demonstrates positivity for complement components (C3d) with negative IgG, reflecting the complement-mediated rather than IgG-mediated mechanism of erythrocyte destruction [3]. A consequence of this pattern, central to the present case, is that a DAT performed with an anti-IgG reagent alone will be negative in CAD.

The clinical presentation of CAD is heterogeneous. Many patients experience anemia, fatigue, jaundice, and cold-induced acrocyanosis or Raynaud-like phenomena, but early disease may manifest with subtle or nonspecific laboratory abnormalities. Isolated hyperbilirubinemia, reflecting increased erythrocyte turnover and heme catabolism, may be the initial and dominant finding. This presentation is poorly characterized in the literature: standard reviews of CAD note jaundice as a feature of established disease but do not describe isolated hyperbilirubinemia as a presenting abnormality preceding clinically apparent anemia, and we identified no prior report in which it drove a prolonged hepatobiliary evaluation [4]. When anemia is mild, cold-induced symptoms are absent, and bilirubin is not fractionated, the hemolytic etiology may go unrecognized for an extended period.

Most cases of CAD occurring without an identifiable secondary trigger are associated with an underlying clonal B-cell lymphoproliferative disorder, most commonly lymphoplasmacytic lymphoma or a related indolent B-cell neoplasm, which serves as the source of the pathogenic IgM autoantibody. This entity is distinguished from secondary cold agglutinin syndrome, in which cold agglutinins arise transiently in the setting of infection, most classically Mycoplasma pneumoniae or Epstein-Barr virus (EBV), or an aggressive lymphoma [1,4]. CAD has only rarely been described in association with myelodysplastic syndrome (MDS) [5-7]. MDS with isolated del(5q) is itself a distinct, genetically defined entity recognized in both the fifth-edition World Health Organization classification and the International Consensus Classification (ICC) 2022 [8,9]. It is characterized by an interstitial deletion of the long arm of chromosome 5 as the sole cytogenetic abnormality, macrocytic anemia with hypolobated megakaryocytes, frequently preserved or elevated platelet counts, a generally indolent course, and a distinctive sensitivity to lenalidomide [10]. Comprehensive bone marrow evaluation is therefore important in patients presenting with unexplained complement-mediated hemolytic anemia because the underlying marrow finding may be unexpected.

Therapeutic options for CAD were historically limited. Corticosteroids and splenectomy are largely ineffective because hemolysis is complement-driven rather than Fc-mediated. Rituximab-based therapy has established activity, although responses to monotherapy are predominantly partial and of limited duration [11]. The development of targeted complement inhibition has represented a significant advance. Sutimlimab, a humanized monoclonal antibody that selectively inhibits complement protein C1s and blocks activation of the classical complement cascade, received regulatory approval for CAD in 2022 and has demonstrated rapid improvement in hemoglobin and hemolysis markers in clinical trials [12,13].

We report a case of CAD that presented as unexplained hyperbilirubinemia and was investigated as hepatobiliary disease for seven and a half months, then as Coombs-negative hemolytic anemia, before the diagnosis was established at nine months. Bone marrow evaluation performed during that workup identified MDS with isolated del(5q) alongside a small IgM-kappa monoclonal protein, a combination that, to our knowledge, has not previously been reported. Treatment with sutimlimab produced rapid and sustained hematologic improvement.

## Case presentation

Presentation and initial evaluation (week 0)

A 75-year-old woman was evaluated for jaundice with hyperbilirubinemia identified on routine laboratory testing. No history of autoimmune disease or recurrent or recent infection was documented, and no iron-containing supplement, herbal preparation, or hepatotoxic medication was recorded. She had received no red blood cell transfusions at any point in her prior care. Abdominal pain, pruritus, dark urine, and acholic stools were absent. Cold-induced acrocyanosis and Raynaud-like symptoms were not documented at presentation or at any point during the subsequent evaluation, and their absence contributed to the delay in considering a cold-antibody process.

Total bilirubin was 5.3 mg/dL (reference range 0.2-1.2 mg/dL), with normal hepatic enzymes: alkaline phosphatase 51 U/L (reference range 40-130 U/L), aspartate aminotransferase (AST) 25 U/L (reference range 10-40 U/L), and alanine aminotransferase (ALT) 13 U/L (reference range 7-52 U/L). Bilirubin was not fractionated. Viral hepatitis serologies, including hepatitis A IgM, hepatitis B surface antigen, hepatitis B core IgM, and hepatitis C antibody, were nonreactive. A complete blood count was obtained but was reported as hemolyzed, and results could not be obtained; in retrospect, this was the first of several specimen abnormalities attributable to cold agglutinins, and it meant that no interpretable hemoglobin was available at presentation.

Because hyperbilirubinemia was isolated and unaccompanied by transaminase or alkaline phosphatase elevation, the working differential comprised extrahepatic biliary obstruction, an infiltrative or cholestatic hepatic process such as primary biliary cholangitis, autoimmune hepatitis, a metabolic liver disease such as hereditary hemochromatosis or Wilson disease, drug-induced liver injury, and inherited disorders of bilirubin conjugation such as Gilbert or Crigler-Najjar syndrome. Hemolysis was not considered, and bilirubin was not fractionated on this or any subsequent specimen until week 33.

Interval course (weeks 4-27)

At week 4, repeat testing showed persistent hyperbilirubinemia with total bilirubin of 3.8 mg/dL and normal liver chemistries (alkaline phosphatase 49 U/L, AST 23 U/L, ALT 12 U/L).

Over the ensuing five months, the patient remained under outpatient evaluation for presumed hepatobiliary disease, monitored with serial laboratory testing. She received no disease-directed therapy during this interval, specifically no corticosteroids, immunosuppressants, transfusions, or iron-containing supplements, so the biochemical trajectory across these months reflects the untreated natural history. She was managed entirely as an outpatient, and no constitutional symptoms, fever, weight loss, night sweats, or lymphadenopathy were documented; the first symptom recorded as attributable to anemia was the dyspnea that prompted emergency evaluation at week 40. Interval symptoms were not systematically documented in the record available for retrospective review, which is acknowledged as a limitation.

At week 23, total bilirubin had risen to 6.2 mg/dL, with liver enzymes again normal, and the first interpretable complete blood count of the evaluation revealed anemia: hemoglobin 9.9 g/dL, red blood cell count 2.97 × 10¹²/L, hematocrit 28.6%, mean corpuscular volume (MCV) 96.3 fL, mean corpuscular hemoglobin (MCH) 33.3 pg (elevated), mean corpuscular hemoglobin concentration (MCHC) 34.6 g/dL, and red cell distribution width (RDW) 16.0% (elevated). The platelet count was elevated at 487 × 10⁹/L, with a mean platelet volume of 12.8 fL. The laboratory appended a comment that the specimen had been prewarmed to 37°C in order to obtain results, with cold agglutinin or cryoglobulin suspected. This was an explicit laboratory flag for cold agglutinins, issued ten weeks before hemolysis was recognized and thirteen weeks before hematology was consulted; its significance was not appreciated at the time. In retrospect, the concurrent thrombocytosis was also a clue to the marrow disorder that would be identified thirteen weeks later, since preserved or elevated platelet counts are characteristic of MDS with isolated del(5q) [10].

At week 25, total bilirubin was 7.8 mg/dL, and transaminases were elevated for the first and only time in the entire course (AST 74 U/L, ALT 154 U/L). Iron studies were markedly abnormal: serum iron 299 µg/dL (reference range 50-170 µg/dL), total iron-binding capacity 209 µg/dL, transferrin saturation 97% (reference range 20-50%), and ferritin 755 ng/mL (elevated). The patient had received no red blood cell transfusions at any point in her care to this date, and none would be given until day 0 of treatment fifteen weeks later, so transfusional iron loading can be excluded with certainty. These transient biochemical abnormalities sustained concern for a primary hepatocellular process and raised hereditary hemochromatosis as a consideration.

At week 27, right upper quadrant ultrasound demonstrated a normal-caliber biliary tree without intrahepatic or extrahepatic ductal dilatation, no cholelithiasis or choledocholithiasis, normal hepatic echotexture and contour, and no focal hepatic lesion.

Recognition of hemolysis (week 33)

At week 33, the patient was referred to gastroenterology. An extended hepatologic workup was unrevealing: antimitochondrial antibody M2 IgG negative, soluble liver antigen antibody 1.8 (within reference range), liver-kidney microsomal antibody IgG less than 1:20, antinuclear antibody negative, ceruloplasmin negative, and repeat viral hepatitis serologies nonreactive. Prothrombin time was normal. EBV serology was reported as positive; the specific antibody profile is not recoverable from the available record, a limitation we address in the Discussion. Iron studies remained abnormal, with serum iron 298 µg/dL, total iron-binding capacity 347 µg/dL, transferrin saturation 85.9%, and ferritin 378 ng/mL.

Testing obtained at the same visit provided the first biochemical evidence of hemolysis. LDH was elevated at 426 U/L (reference range 140-280 U/L), haptoglobin was markedly decreased at less than 10 mg/dL (reference range 30-200 mg/dL), and reticulocytosis was present at 4.5% (reference range 0.5-2.5%), with an absolute reticulocyte count of 120 × 10⁹/L. Total bilirubin was 8.9 mg/dL, and on the first fractionated specimen obtained in the thirty-three weeks since presentation, direct bilirubin measured only 0.9 mg/dL, with alkaline phosphatase 46 U/L, AST 38 U/L, and ALT 14 U/L. The predominantly indirect pattern was inconsistent with biliary obstruction or hepatocellular injury and consistent with hemolysis. Because fractionation had not been performed on any earlier specimen, this pattern had been unavailable during the preceding seven and a half months of evaluation.

Coombs-negative hemolytic anemia and hematologic referral (week 36)

Laboratory studies obtained in advance of the hematology consultation at week 36 confirmed worsening anemia and hemolysis: red blood cell count 2.62 × 10¹²/L, hemoglobin 8.6 g/dL, hematocrit 27.3%, MCV 104.2 fL, and RDW 15.5%; total bilirubin 7.2 mg/dL with conjugated bilirubin 0.5 mg/dL, again predominantly indirect, with alkaline phosphatase 51 U/L, AST 29 U/L, and ALT 14 U/L; haptoglobin less than 10 mg/dL; and antinuclear antibody again negative.

A DAT was performed on this specimen using an anti-IgG reagent alone and was negative. The patient was accordingly referred to hematology with a working diagnosis of Coombs-negative hemolytic anemia. This single result, obtained without a complement-specific reagent, was the principal reason the diagnosis was delayed by a further three weeks after hemolysis had been established.

At the hematology consultation later the same week, laboratory studies showed red blood cell count 2.67 × 10¹²/L, hemoglobin 9.2 g/dL, hematocrit 25.5%, MCV 95.5 fL, MCH 34.5 pg (elevated), and MCHC 36.1 g/dL (elevated); reticulocytes 6.4% with an absolute reticulocyte count of 170 × 10⁹/L; and LDH 444 U/L. An extended hemolytic workup excluded alternative mechanisms: paroxysmal nocturnal hemoglobinuria flow cytometry detected no clone, glucose-6-phosphate dehydrogenase activity was normal at 11.1 U/g Hb, pyruvate kinase activity was elevated at 17.2 U/g Hb rather than deficient, and vitamin B12 (532 pg/mL) and serum folate (5.7 ng/mL) were normal, excluding megaloblastic causes of the macrocytosis.

Serum protein electrophoresis showed albumin 3.81 g/dL, alpha-1 globulin 0.31 g/dL, alpha-2 globulin 0.89 g/dL, beta globulin 0.90 g/dL, and gamma globulin 1.39 g/dL, with no definitive monoclonal protein identified and a quantified M-protein concentration of 0.00 g/dL. Serum immunofixation nevertheless identified a small IgM-kappa monoclonal protein, with kappa free light chain 25.5 mg/L (elevated), lambda free light chain 16.8 mg/L (normal), and a kappa-to-lambda ratio of 1.52. A prednisone taper was initiated and produced minimal improvement in hemolysis markers; the leukocytosis documented over the ensuing three weeks (white blood cell counts of 17.39, 15.70, 13.79, and 13.31 × 10⁹/L with absolute neutrophil counts of 13.04, 9.91, 10.62, and 8.35 × 10⁹/L) is attributable to corticosteroid therapy.

Bone marrow evaluation (week 36)

Bone marrow aspiration and biopsy were undertaken to define the underlying clonal process. The marrow was variably cellular with erythroid-predominant trilineage hematopoiesis; a lymphoid aggregate was present and favored to be benign, and stainable iron was present. Immunohistochemistry for CD61 performed on three blocks showed no overt increase in megakaryocytes but demonstrated occasional small, hypolobated megakaryocytic forms, the morphologic hallmark of MDS with isolated del(5q), although these were focal rather than prominent.

Flow cytometry showed that 9% of total events had the CD45 and light-scatter properties of lymphocytes, comprising T cells (79%, CD4:CD8 ratio 1.13), natural killer cells (1%), and polytypic B cells (19%), with granulocytic elements accounting for 71% of events and no increase in CD34-positive blasts. CD3, CD4, CD5, CD7, CD8, CD13, CD16/56, CD19, CD34, and CD45 expression were normal, and kappa/lambda expression was polytypic. There was no evidence of a lymphoproliferative disorder.

A concurrent peripheral blood smear, drawn from a specimen that the laboratory again noted had been prewarmed because cold agglutinins were present, demonstrated normocytic anemia with anisopoikilocytosis and nonspecific morphologic findings, normal white blood cell and platelet counts, and no overt dysplasia or circulating blasts. The absence of dysplasia on the peripheral smear does not preclude the diagnosis when marrow dysplasia and the defining cytogenetic lesion are present.

Molecular testing identified a LUC7L2 frameshift mutation (p.E251Kfs*6, NM_001244585.1, c.748_749dupGA) with a variant allele frequency of 36.5%; FLT3 internal tandem duplication was not detected. Conventional cytogenetic analysis revealed 46,XX,del(5)(q13q33)(5)/46,XX(15), that is, an interstitial 5q deletion as the sole abnormality in 5 of 20 metaphases. Interphase fluorescence in situ hybridization (FISH) for del(5q) was not performed, and clone size therefore rests on metaphase analysis alone. In the absence of secondary causes, the findings were reported as compatible with involvement by a myeloid neoplasm, best classified as MDS with del(5q) by ICC 2022 and as myelodysplastic neoplasm with low blasts and 5q deletion by the WHO fifth edition [8,9].

Confirmation of cold agglutinin disease (weeks 37-39)

At week 37, cold agglutinin titers were markedly elevated at greater than 1:1024, and cryoglobulin testing was negative.

At week 39, a complete DAT panel was performed. Polyspecific antihuman globulin was positive at 3+, anti-IgG was negative, and anti-C3b/C3d was strongly positive at 3+, establishing complement-mediated hemolysis and reconciling the earlier negative anti-IgG-only result. Repeat EBV testing at this visit was negative, and Mycoplasma pneumoniae PCR was not detected, excluding the two classic infectious triggers of secondary cold agglutinin syndrome. Complement C4 was low at 8 mg/dL (reference range 14-40 mg/dL), consistent with ongoing classical pathway consumption. The findings established CAD without an identified overt B-cell lymphoproliferative disorder, and sutimlimab was recommended at day 0, day 7, and every two weeks thereafter.

Throughout this period, the automated cell indices reflected persistent in vitro agglutination, with spuriously depressed red blood cell counts and elevated MCH and MCHC (MCHC 36.1-37.5 g/dL) and RDW as high as 18.6%.

At week 40, magnetic resonance imaging of the pancreas performed to complete the hepatobiliary evaluation showed a few scattered pancreatic cystic lesions, the largest measuring 1.2 cm in the uncinate process, most consistent with side-branch intraductal papillary mucinous neoplasms, without ductal obstruction or a mass lesion (Table 1).

**Table 1:** Diagnostic timeline from initial presentation to treatment Week 0 is the date of the first abnormal bilirubin. NA indicates the test was not performed at that timepoint. ALP, alkaline phosphatase; AST, aspartate aminotransferase; ALT, alanine aminotransferase; Hct, hematocrit; MCV, mean corpuscular volume; MCH, mean corpuscular hemoglobin; MCHC, mean corpuscular hemoglobin concentration; RDW, red cell distribution width; MPV, mean platelet volume; TIBC, total iron-binding capacity; AMA, antimitochondrial antibody; LKM, liver-kidney microsomal antibody; SLA, soluble liver antigen; ANA, antinuclear antibody; PNH, paroxysmal nocturnal hemoglobinuria; G6PD, glucose-6-phosphate dehydrogenase; PK, pyruvate kinase; SPEP, serum protein electrophoresis; VAF, variant allele frequency; DAT, direct antiglobulin test; AHG, antihuman globulin; IPMN, intraductal papillary mucinous neoplasm; LDH, lactate dehydrogenase.

Week	Clinical event	Total bilirubin (mg/dL)	Hb (g/dL)	LDH (U/L)	Haptoglobin (mg/dL)	Reticulocyte (%)	Other key findings
0	Jaundice identified; hepatobiliary evaluation begun	5.3	Not resulted	NA	NA	NA	ALP 51, AST 25, ALT 13; hepatitis A, B, C serologies non-reactive; CBC reported as hemolyzed; bilirubin not fractionated
4	Repeat outpatient labs	3.8	NA	NA	NA	NA	ALP 49, AST 23, ALT 12
23	Anemia first documented; first laboratory flag for cold agglutinins	6.2	9.9	NA	NA	NA	Hct 28.6%, MCV 96.3 fL, MCH 33.3 pg, RDW 16.0%, platelets 487 × 10⁹/L, MPV 12.8 fL; specimen prewarmed to 37°C, cold agglutinin or cryoglobulin suspected
25	Transient transaminitis and iron abnormality	7.8	NA	NA	NA	NA	AST 74, ALT 154 U/L; iron 299 µg/dL, TIBC 209, transferrin saturation 97%, ferritin 755 ng/mL; no transfusion given at any prior point
27	Right upper quadrant ultrasound	NA	NA	NA	NA	NA	Normal biliary tree, no ductal dilatation or stones, normal hepatic echotexture, no focal lesion
33	Gastroenterology referral; hemolysis first recognized	8.9 (direct 0.9)	NA	426	<10	4.5 (abs 120 × 10⁹/L)	First fractionated bilirubin; AMA-M2, LKM, SLA, ANA, ceruloplasmin all negative; EBV serology positive; iron 298, TIBC 347, transferrin saturation 85.9%, ferritin 378
36 (pre-consultation)	Anti-IgG-only DAT negative	7.2 (conjugated 0.5)	8.6	NA	<10	NA	MCV 104.2 fL, RDW 15.5%; referred as “Coombs-negative hemolytic anemia”
36 (consultation)	Hematology consultation; bone marrow biopsy; prednisone started	NA	9.2	444	NA	6.4 (abs 170 × 10⁹/L)	MCHC 36.1 g/dL; PNH panel negative, G6PD 11.1 normal, PK 17.2 elevated, B12 532, folate 5.7; SPEP without definitive M-protein (M-protein 0.00 g/dL) but immunofixation showing IgM-kappa; kappa free 25.5, lambda free 16.8, ratio 1.52; marrow diagnostic of MDS with isolated del(5q), LUC7L2 p.E251Kfs*6 (VAF 36.5%), 46,XX,del(5)(q13q33)(5)/46,XX(15); CD61 with occasional small hypolobated megakaryocytes; flow without lymphoproliferative disorder
37	Cold agglutinin titer obtained	NA	9.3	NA	NA	NA	Cold agglutinins >1:1024; cryoglobulins negative; WBC 17.39 × 10⁹/L on prednisone
38	Interval labs	NA	9.7	NA	NA	NA	RDW 18.6%, platelets 403 × 10⁹/L
39	Complement-inclusive DAT; CAD confirmed	NA	9.5	NA	NA	NA	Polyspecific AHG 3+, anti-IgG negative, anti-C3b/C3d 3+; EBV repeat negative; Mycoplasma pneumoniae PCR not detected; C4 8 mg/dL; MCHC 36.4 g/dL
40	Emergency department visit for dyspnea; transfusion and sutimlimab the following day (day 0)	8.5	7.3	579	<10	NA	Hct 20.0%, MCHC 36.5 g/dL; MRI pancreas with side-branch IPMNs only

Clinical deterioration and treatment (week 40, day 0 onward)

At week 40, the patient presented to the emergency department with dyspnea. Hemoglobin had fallen to 7.3 g/dL, with a red blood cell count of 2.17 × 10¹²/L, hematocrit 20.0%, and MCHC 36.5 g/dL; total bilirubin was 8.5 mg/dL, LDH 579 U/L, and haptoglobin less than 10 mg/dL. Transfusion was deferred at that encounter, and the scheduled sutimlimab infusion was advanced to the following morning. The next day, designated day 0, she received two units of packed red blood cells followed by the first dose of sutimlimab. This was the only transfusion she received at any point before or since.

Sutimlimab was continued on day 7 and every two weeks thereafter, and she completed four treatment cycles over the following fifteen weeks without significant adverse events.

The hematologic response was rapid and sustained (Table 2, Figures 1, 2). Hemoglobin rose from the pretransfusion nadir of 7.3 g/dL to 9.5 g/dL on day 6; because two units of packed red blood cells were given immediately before the first infusion, this earliest increment cannot be attributed to sutimlimab alone. Hemoglobin continued to rise thereafter with no further transfusions, reaching 10.6 g/dL on day 13 and 11.9 g/dL on day 20, and entering the reference range at 13.0 g/dL by day 33. It peaked at 13.9 g/dL on day 61 and measured 13.2 g/dL at the most recent follow-up on day 103, having remained within the reference range at every measurement from day 33 onward (mean 13.1 g/dL).

**Table 2:** Hematologic response to sutimlimab therapy Reference ranges: hemoglobin 12.0 to 16.0 g/dL; LDH 140 to 280 U/L; reticulocytes 0.5 to 2.5%; total bilirubin 0.2 to 1.2 mg/dL. Two units of packed red blood cells were transfused on day 0 immediately prior to the first sutimlimab infusion, and no transfusion was given at any other point before or after; the day 6 hemoglobin therefore reflects the combined effect of transfusion and treatment. Sutimlimab was administered on day 0 and day 7, then every two weeks. Total bilirubin at day 20 was drawn on day 21. NA indicates the value was not measured at that time point. Serial post-treatment haptoglobin was not measured; haptoglobin was less than 10 mg/dL on every pre-treatment determination and is frequently uninformative for monitoring once suppressed below the assay's limit of detection, and its absence during follow-up is acknowledged as a limitation. LDH, lactate dehydrogenase.

Time after first dose	Hb (g/dL)	LDH (U/L)	Reticulocyte (%)	Absolute reticulocyte (× 10⁹/L)	Total bilirubin (mg/dL)
Day 0 (pre-transfusion nadir)	7.3	579	NA	NA	8.5
Day 6 (week 1)	9.5	628	6.7	199	NA
Day 13 (week 2)	10.6	335	4.0	141	NA
Day 20 (week 3)	11.9	459	2.3	88	2.2 (day 21)
Day 33 (week 5)	13.0	NA	NA	NA	NA
Day 47 (week 7)	13.3	359	1.4	NA	NA
Day 61 (week 9, peak Hb)	13.9	NA	1.6	NA	NA
Day 75 (week 11)	13.0	425	2.0	NA	NA
Day 89 (week 13)	12.4	350	2.1	NA	NA
Day 103 (week 15, most recent)	13.2	289	1.8	NA	NA

**Figure 1:**
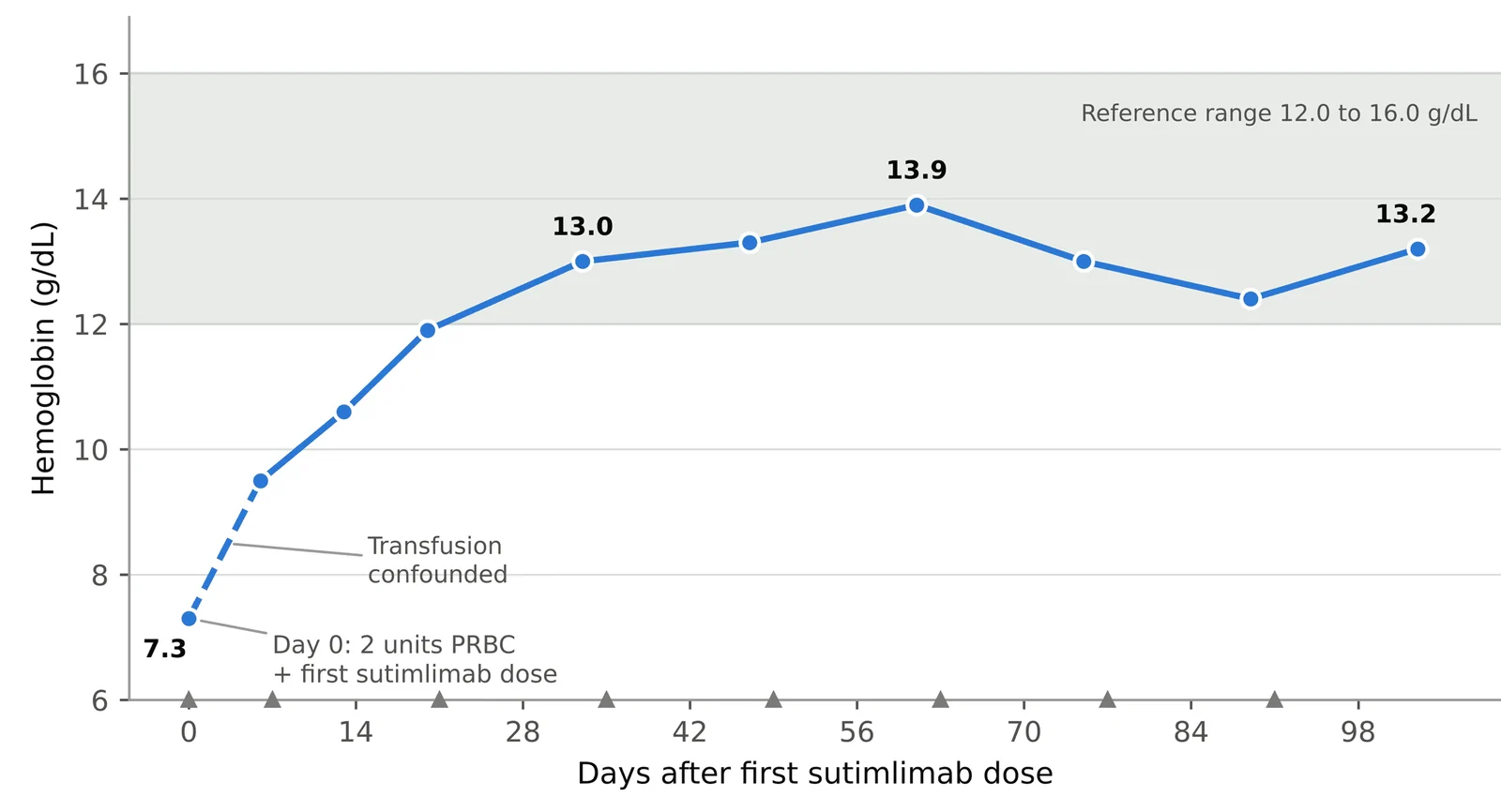
Hemoglobin response to sutimlimab therapy Hemoglobin at days 0, 6, 13, 20, 33, 47, 61, 75, 89, and 103 after the first sutimlimab dose. Triangles on the x-axis mark infusions (day 0, day 7, then every two weeks). The shaded band is the reference range, 12.0 to 16.0 g/dL. Two units of packed red blood cells were transfused on day 0 immediately before the first infusion; the day 0 to day 6 segment is shown as a dashed line because that increment reflects the combined effect of transfusion and treatment and cannot be attributed to sutimlimab alone. Hemoglobin rose from a nadir of 7.3 g/dL, entered the reference range by day 33, peaked at 13.9 g/dL at day 61, and remained within the reference range through day 103. No further transfusions were given.

**Figure 2:**
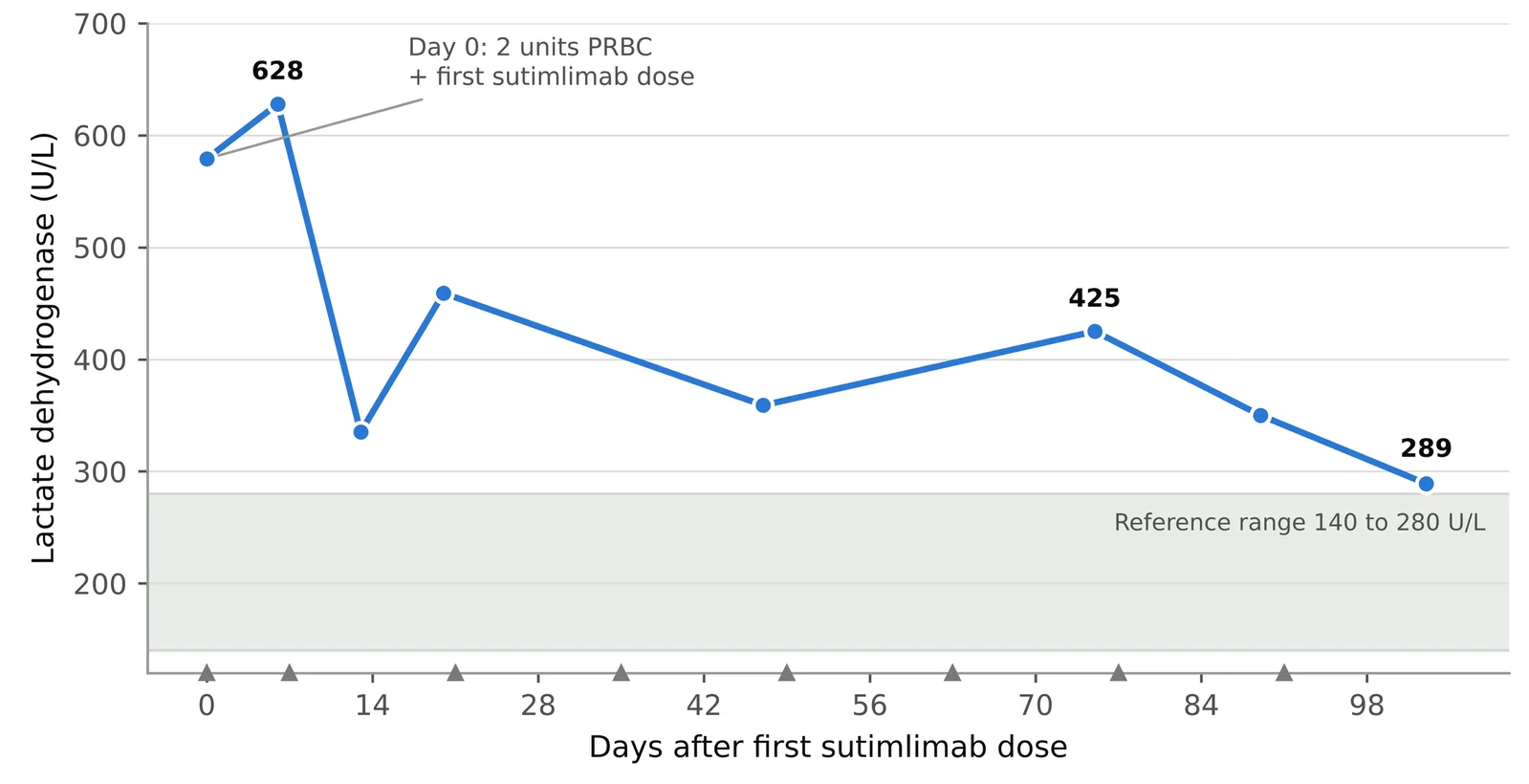
Lactate dehydrogenase (LDH) trend following sutimlimab initiation LDH at the same timepoints as Figure 1 and Table 2. Triangles on the x-axis mark infusions (day 0, day 7, then every two weeks). The shaded band is the reference range, 140 to 280 U/L. LDH rose transiently to a peri-transfusion peak of 628 U/L at day 6, then declined to 289 U/L at day 103, remaining marginally above the upper limit of normal, with intermittent fluctuations including a rise to 425 U/L at day 75 consistent with residual low-grade hemolysis. LDH was not measured at days 33 and 61.

Markers of hemolysis improved concurrently. LDH rose transiently to a peritransfusion peak of 628 U/L on day 6, then fell to 335 U/L on day 13 and to 289 U/L on day 103, with intermittent fluctuations, including a rise to 425 U/L on day 75, consistent with residual low-grade hemolysis. The reticulocyte percentage, 6.4% before treatment and 6.7% on day 6, fell to 2.3% by day 20 and remained between 1.4% and 2.1% thereafter, measuring 1.8% on day 103 and thus within the reference range. Total bilirubin improved substantially from 8.5 mg/dL to 2.2 mg/dL by day 21, remaining modestly above the reference range. The spurious red cell indices resolved in parallel, with MCHC falling from 37.5 g/dL on day 13 to 34.0 g/dL on day 33, MCV from 100.4 to 90.1 fL, and RDW from 18.6% to 13.0%, consistent with resolution of in vitro agglutination.

The patient required no further transfusions at any point during follow-up. Cold-induced symptoms were not documented before or during treatment; formal symptom assessment after day 21 was limited by the absence of subsequent office visits, with monitoring thereafter conducted through scheduled infusions and laboratory testing.

## Discussion

CAD is a complement-mediated autoimmune hemolytic anemia in which cold-reactive IgM autoantibodies bind erythrocytes at reduced temperatures and activate the classical complement pathway, resulting in C3 fragment deposition on the red cell surface and predominantly extravascular hemolysis within the reticuloendothelial system [1,2]. The characteristic laboratory profile includes anemia, elevated LDH, markedly decreased haptoglobin, reticulocytosis, and a DAT positive for complement but negative for IgG, a pattern that distinguishes CAD from warm autoimmune hemolytic anemia [3,4].

An unusual coexistence, and what it does and does not establish

The principal finding in this case is the coexistence of CAD, a small IgM-kappa monoclonal protein, and MDS with isolated del(5q). We wish to be explicit about the limits of this observation. The del(5q) MDS is demonstrably a clonal myeloid process. An IgM-kappa monoclonal protein in a 75-year-old patient does not by itself establish a second clonal B-cell process, nor does it establish that the paraprotein is the source of the pathogenic cold agglutinin, particularly given that serum protein electrophoresis quantified the M-protein at 0.00 g/dL and the abnormality was detectable only by immunofixation. Conversely, the absence of a demonstrable B-cell lymphoproliferative disorder on marrow morphology and flow cytometry does not exclude a small clonal B-cell population below the limit of detection. We therefore describe an unusual coexistence rather than a causal or clonal relationship, and we do not suggest that the MDS is responsible for the CAD.

To assess whether this combination has been described previously, we searched PubMed/MEDLINE, Embase, Scopus, and Web of Science from database inception through June 2026, without language restrictions, using combinations of the terms "cold agglutinin disease," "cold agglutinin syndrome," "cold autoimmune hemolytic anemia," "myelodysplastic syndrome," "MDS," "del(5q)," "5q deletion," and "5q minus syndrome," supplemented by hand-searching the reference lists of retrieved articles. Non-English reports were assessed by translation of the abstract and, where available, the full text, rather than excluded. Inclusion required a reported case of cold agglutinin-mediated hemolysis in a patient with an established diagnosis of MDS. The search identified five prior reports [6,7,14-16]. In none was the cytogenetic abnormality an isolated del(5q). A single additional report described lenalidomide-associated CAD in a patient with del(5q) MDS [17]; because our patient never received lenalidomide or any other immunomodulatory agent, that drug-related mechanism does not apply here. To our knowledge, and contingent on the completeness of this search, the present case is therefore the first report of CAD arising in association with MDS with isolated del(5q) specifically, as distinct from the broader and previously described category of cold agglutinin-mediated hemolysis in MDS.

Three explanations for the coexistence are possible and cannot be distinguished with the available data: the cold agglutinin may originate from an occult clonal B-cell population independent of the del(5q) myeloid clone; autoantibody production may reflect the dysregulated immune environment that accompanies myeloid neoplasia, given that autoimmune phenomena occur in approximately 15% of patients with MDS across large series [5]; or the association may be coincidental. Notably, the LUC7L2 frameshift mutation identified here is located at 7q34, and recurrent LUC7L2 mutation or loss in myeloid neoplasms is associated with monosomy 7 and del(7q) rather than with del(5q) [18]. We cite it only as a recurrently mutated spliceosomal gene supporting the molecular diagnosis of MDS, and we make no claim of a mechanistic link to autoantibody production. Lenalidomide, the standard therapy for symptomatic anemia in del(5q) MDS [10], was not administered, which allowed management to be directed at the hemolytic process and also removes lenalidomide-induced cold agglutinin formation as a confounder.

A presentation that mimics hepatobiliary disease

The dominant initial abnormality was isolated hyperbilirubinemia rather than overt anemia, and evaluation was directed toward hepatobiliary disease for seven and a half months. Hyperbilirubinemia in CAD arises from accelerated erythrocyte turnover and heme catabolism and produces a predominantly indirect pattern once bilirubin is fractionated, as demonstrated here on both fractionated specimens: direct bilirubin was 0.9 mg/dL against a total of 8.9 mg/dL at week 33, and conjugated bilirubin was 0.5 mg/dL against a total of 7.2 mg/dL at week 36. Critically, no specimen was fractionated before week 33. The single inexpensive test that would have redirected the evaluation was simply never ordered during the preceding thirty-three weeks, and hemolysis was recognized at the same visit at which fractionation was finally performed. Table 1 makes the cost of that omission explicit: total bilirubin was measured on five occasions and rose from 5.3 to 8.9 mg/dL before anyone asked how much of it was conjugated.

Several features sustained the hepatic hypothesis. The transaminase elevation at week 25 was isolated, transient, and never recurred across more than a dozen subsequent panels. The markedly elevated transferrin saturation of 97%, with ferritin of 755 ng/mL, is not explained by hemolytic iron release alone. In this patient, it is best understood as the combined effect of iron released by ongoing hemolysis and ineffective erythropoiesis intrinsic to MDS, both of which increase erythroferrone-mediated suppression of hepcidin and thereby raise transferrin saturation [19]. Transfusional iron loading can be excluded with certainty because the patient's only transfusion in her entire course was given on day 0 of treatment, fifteen weeks after the abnormal iron studies. Marrow examination confirmed that stainable iron was present rather than depleted. Clinicians should maintain a low threshold for fractionating bilirubin and for measuring LDH, haptoglobin, and reticulocyte count in any patient with unexplained hyperbilirubinemia, particularly when hepatobiliary imaging and serologies are unrevealing [4].

Two immunohematologic pitfalls

The second and more consequential pitfall was diagnostic rather than preanalytical. A DAT performed at week 36 with an anti-IgG reagent alone was negative, and the patient was referred to hematology with a working diagnosis of Coombs-negative hemolytic anemia, a framing that directed the subsequent workup toward paroxysmal nocturnal hemoglobinuria, enzymopathies, and marrow disease rather than toward complement-mediated immune hemolysis. Only when a complete panel including an anti-C3b/C3d reagent was performed at week 39 did the mechanism become apparent, with polyspecific antihuman globulin and anti-C3b/C3d both positive at 3+ while anti-IgG remained negative. Because CAD is by definition complement-mediated and IgG-negative, an anti-IgG-only DAT is expected to be negative in this disease; a negative result obtained without a complement-specific reagent therefore excludes nothing and should not be reported or interpreted as a negative Coombs test [3]. In a patient with unexplained hemolysis, the DAT must be complement-inclusive.

The first pitfall was preanalytical and, in retrospect, was flagged repeatedly by the laboratory itself (Table 1). The complete blood count at presentation was reported as hemolyzed, and results could not be obtained. Ten weeks before hemolysis was recognized, the laboratory appended an explicit comment that the specimen required prewarming to 37°C and that cold agglutinin or cryoglobulin was suspected, and it repeated that comment at week 36. Beyond these narrative flags, the automated indices themselves carried the signature of in vitro agglutination: a spuriously depressed red blood cell count with elevated MCH and MCHC, the latter reaching 37.5 g/dL, and RDW as high as 18.6%. An MCHC above the physiologic ceiling is not biologically possible and should prompt consideration of agglutination, cold agglutinins, lipemia, or spherocytosis. That these indices normalized during treatment, with MCHC falling to 34.0 g/dL, MCV from 100.4 to 90.1 fL, and RDW to 13.0%, supports agglutination as their cause. Recognition of these signals, with prompt use of prewarmed specimens and complement-inclusive reagents, is essential to avoid missing complement-mediated hemolysis.

Distinguishing primary disease from secondary cold agglutinin syndrome

The positive EBV serology obtained at week 33 introduced an additional consideration because EBV is a well-recognized trigger of secondary cold agglutinin syndrome. Repeat testing six weeks later was negative, and Mycoplasma pneumoniae PCR performed at the same visit was likewise negative, excluding the two classic infectious triggers. We note that the specific EBV antibody profile at each time point is not recoverable from the available record, so we cannot state which serologic component converted; this constrains interpretation and is reflected in the cautious conclusion below. The broader picture, including a cold agglutinin titer above 1:1024, low C4 indicating sustained classical pathway consumption, the IgM-kappa monoclonal protein, and clonal marrow findings, favored a persistent rather than a transient postinfectious process [1,4]. We do not characterize the initial serology as a false positive, which the available data cannot establish; we conclude only that the findings did not support ongoing EBV-associated secondary cold agglutinin syndrome.

Severity of the anemia and its dual mechanism

The anemia in this patient was severe and of dual origin. Peripheral consumption from complement-mediated hemolysis was superimposed on impaired central production from MDS, in which ineffective erythropoiesis limits the compensatory marrow response. The absolute reticulocyte count peaked at 199 × 10⁹/L and was 170 × 10⁹/L at a hemoglobin of 9.2 g/dL, a response that is modest for the degree of anemia and hemolysis present and consistent with constrained marrow reserve. This combination accounts for the progressive, symptomatic decline to a hemoglobin of 7.3 g/dL with dyspnea prompting emergency evaluation, despite a hemolytic rate that in a patient with intact erythropoiesis might have been compensated. Recognizing the dual mechanism is clinically consequential: where marrow reserve is limited, ongoing complement-mediated destruction is poorly tolerated and can become life-threatening more rapidly than the hemolytic rate alone would suggest, which argues for prompt rather than stepwise escalation to effective complement inhibition.

Treatment response

Corticosteroids are generally ineffective in CAD because hemolysis is driven by complement activation rather than by the Fc receptor-mediated clearance that steroids target in warm autoimmune hemolytic anemia [4]; the minimal response to the prednisone taper here is consistent with that expectation, and the only clear effect of corticosteroid therapy was transient leukocytosis. Rituximab monotherapy achieves response rates of approximately 50-54%, although responses are predominantly partial and the median duration is limited to approximately 11-12 months [11]; combinations with fludarabine or bendamustine achieve higher and more durable responses at the cost of greater toxicity [20,21]. Sutimlimab, a humanized monoclonal antibody targeting complement C1s, selectively inhibits the classical cascade upstream of C3 activation, preventing complement deposition and halting hemolysis [12,13]. In the pivotal CARDINAL trial, sutimlimab produced rapid increases in hemoglobin and improvement in hemolysis markers within the first week of treatment [12].

The response observed here was consistent with that experience, with an important interpretive caveat. Because two units of packed red blood cells were transfused immediately before the first infusion, the rise in hemoglobin from 7.3 to 9.5 g/dL on day 6 cannot be attributed to sutimlimab alone. The evidence for a treatment effect rests instead on the sustained subsequent gain to 13.0 g/dL by day 33 and stability within the reference range at every measurement through day 103, achieved without any further transfusions (Figure 1, Table 2), together with the fall in LDH (Figure 2), the return of the reticulocyte count to the reference range, the substantial improvement in bilirubin, and the resolution of the agglutination artifact in the automated red cell indices. Not all markers reached the reference range: LDH remained marginally elevated at 289 U/L on day 103 against an upper limit of 280 U/L, and total bilirubin remained at 2.2 mg/dL on day 21 against an upper limit of 1.2 mg/dL. The response is therefore best described as near-normalization of LDH and substantial improvement in bilirubin rather than complete biochemical normalization. The more gradual and fluctuating decline in LDH relative to the prompt hemoglobin recovery, including the transient rise to 425 U/L on day 75, likely reflects cessation of overt red cell destruction alongside residual low-grade complement activity and should not be misinterpreted as treatment failure.

Limitations

Several limitations should be noted. First, the del(5q) clone was identified in 5 of 20 metaphases by conventional karyotyping, and interphase FISH was not performed; clone size therefore rests on metaphase analysis alone and may be underestimated. Second, no clonality studies, such as immunoglobulin heavy chain gene rearrangement or MYD88 L265P testing, were performed to determine whether the IgM-kappa paraprotein derives from a clonal B-cell population, and the cold agglutinin was not demonstrated to be the monoclonal IgM; the pathogenic source of the autoantibody therefore remains unestablished. Third, the dysplastic megakaryocytic changes, while present, were focal, with occasional small, hypolobated forms and no overt increase in megakaryocyte number, so the diagnosis rests substantially on the defining cytogenetic abnormality in the absence of secondary causes. Fourth, serial post-treatment haptoglobin was not measured, which constrains assessment of the completeness of the hemolytic response, and LDH and reticulocytes were not measured at every plotted time point. Fifth, the specific EBV antibody profile at the positive and subsequent negative determinations is not recoverable from the available record, so the significance of the initial positive result cannot be fully adjudicated. Sixth, this is a retrospective review of the clinical record, and interval symptoms across the observation period were not systematically documented; follow-up extends to day 103, so durability beyond fifteen weeks is unknown, and symptom assessment after day 21 was limited by the absence of subsequent office visits. Finally, this is a single case, and a novelty claim based on a literature search is necessarily contingent on the completeness of that search and on publication practices.

## Conclusions

CAD is a rare complement-mediated hemolytic anemia that may present insidiously and mimic hepatobiliary disease when hyperbilirubinemia predominates, anemia is initially unrecognized, and cold-induced symptoms are absent. This case illustrates two failure modes that together produced a nine-month delay. Bilirubin was never fractionated during the first thirty-three weeks, so the indirect, hemolytic pattern remained undetected throughout an extensive hepatobiliary workup. Then, once hemolysis was established, a DAT performed with an anti-IgG reagent alone returned a negative result, and the patient was labeled as having Coombs-negative hemolytic anemia, a result that excludes nothing in a disease that is by definition IgG-negative and complement-mediated. Laboratory signals of cold agglutinin interference, including prewarming comments and a physiologically impossible mean corpuscular hemoglobin concentration, had been present for months. A systematic approach incorporating bilirubin fractionation, hemolysis markers, attention to spurious red cell indices, and a prewarmed, complement-inclusive DAT is therefore essential.

The concurrent identification of MDS with isolated del(5q) alongside a small IgM-kappa monoclonal protein represents, to our knowledge, the first reported coexistence of this kind with CAD. We describe it as a coexistence rather than a causal association because the source of the pathogenic cold agglutinin was not established, and it nonetheless underscores the value of bone marrow evaluation in unexplained complement-mediated hemolysis. Where hemolysis is superimposed on the impaired erythropoiesis of MDS, the resulting anemia can be severe and poorly compensated; here, targeted complement inhibition with sutimlimab produced a rapid and sustained transfusion-independent hematologic response, supporting its role in the management of complement-mediated CAD.
